# A comprehensive review for machine learning on neuroimaging in obsessive-compulsive disorder

**DOI:** 10.3389/fnhum.2023.1280512

**Published:** 2023-11-01

**Authors:** Xuanyi Li, Qiang Kang, Hanxing Gu

**Affiliations:** ^1^Department of Radiology, Shengjing Hospital of China Medical University, Shenyang, Liaoning, China; ^2^Department of Radiology, Xing’an League People’s Hospital of Inner Mongolia, Mongolia, China; ^3^Department of Geriatric Psychiatry, Qingdao Mental Health Center, Qingdao, Shandong, China

**Keywords:** neuroimaging, MRI, machine learning, obsessive-compulsive disorder, AI

## Abstract

Obsessive-compulsive disorder (OCD) is a common mental disease, which can exist as a separate disease or become one of the symptoms of other mental diseases. With the development of society, statistically, the incidence rate of obsessive-compulsive disorder has been increasing year by year. At present, in the diagnosis and treatment of OCD, The clinical performance of patients measured by scales is no longer the only quantitative indicator. Clinical workers and researchers are committed to using neuroimaging to explore the relationship between changes in patient neurological function and obsessive-compulsive disorder. Through machine learning and artificial learning, medical information in neuroimaging can be better displayed. In this article, we discuss recent advancements in artificial intelligence related to neuroimaging in the context of Obsessive-Compulsive Disorder.

## 1. Introduction

### 1.1. The current situation of obsessive-compulsive disorder

#### 1.1.1. Epidemiology of obsessive-compulsive disorder

Obsessive-Compulsive Disorder (OCD) is a severe mental disorder characterized primarily by frequent, uncontrollable obsessive thoughts and compulsive behaviors. These obsessive thoughts are often referred to as obsessions, while the corresponding compulsive behaviors are seen as coping mechanisms aimed at alleviating the distress caused by these thoughts. These obsessions and compulsions can significantly disrupt a patient’s daily life, affecting their social interactions, academic performance, work, and family life (Fontenelle et al., 2006). The exact pathogenesis of obsessive-compulsive disorder (OCD) remains incompletely understood, but research suggests that it involves complex neurobiological and genetic factors. Many studies emphasize the role of imbalances in neurotransmitters such as glutamate, serotonin, and dopamine in the onset of OCD. Additionally, environmental factors, life events, and cognitive factors may also play a role in the development of OCD. OCD is a global issue, affecting a substantial portion of the population. According to statistical data, the prevalence of OCD worldwide is estimated to be around 2–3%, with no significant differences observed between different countries and regions (Robbins et al., 2019). The onset of OCD typically occurs in adolescence to early adulthood, although cases with later onset are also reported. The ratio of males to females affected by OCD is roughly equal (Ruscio et al., 2010). Obsessive-compulsive disorder (OCD) significantly impacts the quality of life for those affected. Many patients report substantial distress and suffering due to their OCD symptoms, which can even lead to complications such as depression and anxiety. Because of the recurrent and long-lasting nature of the symptoms, patients often feel fatigued and helpless. In severe cases, OCD can prevent individuals from engaging in normal social activities and may even result in a loss of social functioning (Bloch et al., 2008). The diagnosis of obsessive-compulsive disorder (OCD) is typically achieved through clinical assessment and psychological testing. In recent years, neuroimaging and related fields of artificial intelligence have also shown promising results in diagnosing OCD. The World Health Organization (WHO) and the Diagnostic and Statistical Manual of Mental Disorders (DSM) provide criteria for diagnosing OCD. Clinicians often determine the diagnosis by inquiring about the patient’s symptoms, family history, and life events. While there is currently no definitive cure for OCD, there are multiple treatment options available to help alleviate symptoms and improve the quality of life for patients. Cognitive Behavioral Therapy (CBT) is considered the first-line treatment for OCD. CBT works by helping patients recognize and modify negative thought patterns and behavioral patterns to reduce OCD symptoms. For patients with more severe symptoms, medication therapy may be necessary. Commonly used medications include Selective Serotonin Reuptake Inhibitors (SSRIs) and Tricyclic Antidepressants (TCAs). In some cases, a combination of psychotherapy and medication therapy might yield better treatment outcomes (Frare et al., 2004).

In summary, obsessive-compulsive disorder (OCD) is a common and severe mental disorder that affects millions of people worldwide. While our understanding of its pathogenesis remains incomplete, factors such as neurobiology, genetics, environmental influences, and cognitive factors may all play a role in its development. Although there is currently no definitive cure, approaches like cognitive-behavioral therapy and medication therapy can help patients alleviate symptoms and improve their quality of life.

#### 1.1.2. Diagnosis and evaluation of obsessive-compulsive disorder

Due to the prevalence of OCD and its significant impact on patients’ social functioning and quality of life, a clear diagnosis is particularly important. The diagnosis of OCD is primarily conducted through neuroimaging, clinical assessment, and self-report symptom scales. The diagnostic criteria for OCD are mainly based on internationally recognized classification systems for mental disorders, namely the Diagnostic and Statistical Manual of Mental Disorders (DSM) and the International Classification of Diseases (ICD) by the World Health Organization. In DSM-5, the criteria for diagnosing OCD require that the patient must meet the following conditions: (A) Presence of obsessions, compulsions, or both. (B) Obsessions or compulsions are time-consuming or cause significant distress with clinical significance or result in impairment in social, occupational, or other important areas of functioning. (C) Obsessive-compulsive symptoms are not attributable to the physiological effects of a substance or another medical condition. (D) The disorder is not better explained by another mental disorder. According to the ICD-11 (draft): (1) The thoughts or impulses must be the patient’s own. (2) At least one thought or action must be resisted futilely by the patient, even though they may no longer resist other symptoms. (3) The idea of carrying out the action itself should be unpleasant. (4) Thoughts, images, or impulses must recur repeatedly and be unpleasant. These criteria serve as essential guidelines for clinicians to diagnose OCD accurately.

Symptom self-assessment scales are tools widely used in the diagnosis of obsessive-compulsive disorder (OCD). Common diagnostic tools include the Yale-Brown Obsessive-Compulsive Scale (Y-BOCS) and the Obsessive-Compulsive Inventory-Revised (OCI-R) (American Psychiatric Association, 2013), among others. The most commonly used one is the Yale-Brown Obsessive-Compulsive Scale. The Yale-Brown Obsessive-Compulsive Scale (Goodman et al., 1989) is a standardized clinician-rated assessment scale widely employed in cognitive, behavioral, and pharmacological treatment trials. It consists of a series of questions covering the frequency, severity, and interference level of various OCD symptoms. Patients rate each question based on their own experiences, helping clinicians understand the severity of their OCD symptoms. Other scales include the Maudsley Obsessional-Compulsive Inventory (MOCI), Zung-Ferris Obsessive-Compulsive Symptom Scale (ZF-OCS) (Foa et al., 1998), and more. Despite having clear diagnostic criteria, diagnosing OCD remains a complex process. Due to the diversity of symptoms and presentations among patients, as well as potential overlap with other mental disorders, a comprehensive assessment by experienced physicians and psychologists is necessary. The development of neuroimaging techniques has provided new avenues for the diagnosis of OCD, but their application requires further research and validation. The combination of clinical assessments, symptom self-assessment scales, and neuroimaging examinations can lead to a more accurate diagnosis of OCD and provide a basis for individualized treatment plans for patients.

#### 1.1.3. Treatment and prognosis of obsessive-compulsive disorder

An increasing body of evidence suggests that untreated illnesses with longer durations lead to poorer outcomes and prognosis (Fineberg et al., 2019). Therefore, it is crucial for individuals with obsessive-compulsive disorder (OCD) to receive appropriate treatment promptly in order to alleviate suffering and improve functioning.

##### 1.1.3.1. Medication treatment for OCD

Medication treatment for OCD primarily includes antidepressants, antipsychotic drugs, and antiepileptic medications. As a first-line treatment, the long-term use of Selective Serotonin Reuptake Inhibitors (SSRIs) is the most effective. SSRIs such as sertraline, fluoxetine, fluvoxamine, paroxetine, and escitalopram have demonstrated high response rates and positive long-term outcomes (Kaplan and Hollander, 2003). Research has shown that SSRIs can provide significant benefits as early as 2 weeks into treatment (Issari et al., 2016), and lower doses (e.g., escitalopram) can effectively prevent relapse (Fineberg et al., 2007). Clomipramine is a tricyclic antidepressant (TCA) that falls between SSRIs and serotonin-norepinephrine reuptake inhibitors (SNRIs) and has a unique anti-OCD effect (Bontempo et al., 2012). It was the first drug approved for OCD treatment. While clomipramine is effective in treating OCD and intravenous use is effective for refractory OCD, it comes with more common and severe side effects (Association, 2013). The most dangerous clomipramine-related adverse reactions, similar to other TCAs, include seizures and elevated liver enzymes. Serotonin-Norepinephrine Reuptake Inhibitors (SNRIs) like venlafaxine have short-term efficacy in treating OCD similar to clomipramine but with better safety and tolerability (Albert et al., 2002). Agomelatine’s 5-HT2C antagonism mediates its anti-anxiety effect and helps regulate melatonin (MT1 and MT2 receptor antagonism) (De Berardis et al., 2013), contributing to the restoration of circadian rhythms in OCD patients and alleviating some symptoms. Antipsychotic medications like olanzapine (Koran et al., 2005; Li et al., 2005) can be used in combination therapy for refractory OCD, often combined with SSRIs. Research has shown that certain antiepileptic drugs (Önder et al., 2008; Arrojo-Romero et al., 2013), as well as aripiprazole (Soltani et al., 2010), and dextromethorphan (Liddell et al., 2013), have a certain alleviating effect on refractory OCD. These medication options provide a range of choices for treating OCD, and the choice of treatment should be made in consultation with a healthcare provider based on the individual patient’s needs and circumstances.

##### 1.1.3.2. Brain stimulation

Increasing evidence suggests that OCD is associated with dysfunction in the orbitofronto-striato-pallido-thalamic circuit. This circuit includes the dorsolateral prefrontal cortex (DLPFC), orbitofrontal cortex (OFC), medial prefrontal cortex (MPFC), anterior cingulate cortex (ACC), supplementary motor area (SMA), and basal ganglia (Del Casale et al., 2011; Fineberg et al., 2011). Severe treatment-resistant OCD may benefit from non-invasive techniques, including repetitive transcranial magnetic stimulation (rTMS) (Saba et al., 2015), deep transcranial magnetic stimulation (dTMS), and transcranial direct current stimulation (tDCS) (Carmi et al., 2019), as well as invasive deep brain stimulation (DBS) (Blomstedt et al., 2013) and electroconvulsive therapy (ECT) (Bais et al., 2014).

##### 1.1.3.3. Psychological intervention

Cognitive-behavioral therapy (CBT) is the preferred psychological intervention for OCD. It is a structured, short-term, cognitive-oriented psychotherapy method developed by A.T. Beck in the 1960s. Its main focus is on the patient’s irrational cognitive issues, aiming to change the patient’s views and attitudes towards themselves, others, or events to address psychological problems. For children and young people, CBT is prioritized over medication (Uhre et al., 2020). The recommended type of CBT for OCD is Exposure and Response Prevention (ERP). ERP is a therapy that teaches patients to face and tolerate the obsessions that trigger their compulsions and resist taking action. It has shown good therapeutic effects in patients with OCD (Abramowitz, 1997). Most patients experience symptom improvement when treated with medication alone or in combination with cognitive therapy (Skapinakis et al., 2016). In clinical practice, SSRI treatment is often combined with cognitive-behavioral therapy for the treatment of OCD patients (Cottraux et al., 2005). However, even with theoretically appropriate treatment approaches in place, 40–60% of OCD patients still exhibit disabling residual symptoms, indicating the need for innovative drug therapies, different augmentation strategies, and new physical treatment techniques (Pallanti and Quercioli, 2006).

#### 1.1.4. Artificial intelligence in obsessive-compulsive disorder

At present, the application of artificial intelligence in OCD involves all stages of the diagnosis and treatment of OCD. The data used include scales, sensor data and medical image data. Without a doubt, the scale is the most readily available data for assessing OCD. A 2020 study of 400 healthy controls and 200 people diagnosed with obsessive-compulsive disorder added additional items to the Y-BOCS scale to assess patients. The researchers studied OCD by building an artificial neural network (Shahzad et al., 2020). Wearables are also of interest because of the intermittent nature of mental illness. A 2023 study wearing wristbands for children and adolescents ages 8 to 17 with OCD explored the feasibility and acceptability of wearable biosensors to monitor OCD symptoms (Lønfeldt et al., 2023). Some studies have used daily voice and image information to diagnose and track OCD. In a 2022 study involving 47 obsessive-compulsive disorder (OCD) and 17 healthy adolescents, researchers analyzed the subjects’ speech to explore the link between OCD severity and vocal features (Clemmensen et al., 2022). Neuroimaging is highly standardized and easy to quantify, so neuroimaging has been paid more and more attention by researchers and doctors in the diagnosis and treatment of mental diseases. Magnetic resonance imaging (MRI) and electroencephalogram (EEG) are the most commonly used tests in OCD, and almost all neuroimaging-related AI research is based on them (Abd-Alrazaq et al., 2022).

### 1.2. The application of neuroimaging in obsessive-compulsive disorder

In recent years, the use of neuroimaging in the diagnosis of OCD has been increasing. Particularly, techniques like functional magnetic resonance imaging (fMRI) and single-photon emission computed tomography (SPECT) can help reveal the characteristics of brain activity in OCD patients. Through neuroimaging, we can observe abnormal brain activity in regions associated with OCD, providing more biological evidence for the research and treatment of OCD.

#### 1.2.1. Magnetic resonance imaging (MRI)

Functional Magnetic Resonance Imaging (fMRI) technology typically refers to magnetic resonance imaging (MRI) techniques that analyze brain activity using the Blood Oxygenation-Level-Dependent (BOLD) phenomenon. Over the past few decades, neuroimaging research has discovered abnormal structure and function in the cortical-striatal-thalamic-cortical (CSTC) circuitry of OCD patients. Following the induction and provocation of OCD symptoms, functional magnetic resonance imaging (fMRI) has observed corresponding changes in the activation of the orbitofrontal cortex, dorsolateral prefrontal cortex (PFC), and anterior cingulate cortex (ACC), as well as the caudate nucleus, insula, amygdala and border structure (Simon et al., 2010). For example, after using fluvoxamine or behavioral therapy to improve OCD, there is a reduction in symptom-provoked activation in the orbitofrontal cortex, dorsolateral prefrontal cortex, and ACC (Nakao et al., 2005). One study suggested that OCD symptoms were associated with increased activation in the bilateral prefrontal cortex, left insula, right superior frontal gyrus, left caudate nucleus, and right thalamus (Schienle et al., 2005). Conversely, there is research indicating that effective OCD treatments such as selective serotonin reuptake inhibitors (SSRIs), ERP, or deep brain stimulation (DBS) can restore normal activity in the orbitofrontal cortex (OFC) observed in fMRI (Saxena et al., 1999; Mataix-Cols et al., 2003, 2004; Banca et al., 2015). These studies collectively support the role of functional dysregulation in the CSTC circuit in the development of OCD.

#### 1.2.2. Electroencephalogram

An electroencephalogram (EEG) is a graphical representation obtained by amplifying the spontaneous bioelectric potentials of the cerebral cortex of the brain, recorded from the scalp using precise instruments (Biasiucci et al., 2019). It plots electrical potential on the vertical axis and time on the horizontal axis, thus creating a graphical representation of the relationship between electrical potentials and time. EEG is relatively inexpensive, non-invasive, portable, and easy to manage. However, research using EEG to investigate Obsessive-Compulsive Disorder (OCD) is relatively limited compared to fMRI. This might be due to the difficulty in standardizing the positions for signal reception during recording, making it challenging to determine the precise location of the electrophysiological source (Michel and Brunet, 2019). To pinpoint specific locations, low-resolution brain electromagnetic tomography (LORETA) may be required (Dattola et al., 2020). The occurrence of OCD is believed to be associated with excessive activity of erroneous signals in the brain, such as increased amplitudes of error-related negativity (ERN) (Riesel, 2019). Using auditory oddball paradigms can elicit specific P300 waveforms in OCD patients (Sur and Sinha, 2009). Some research suggests that changes in EEG waveforms can predict treatment responses of OCD patients to psychotherapy and medication (Krause et al., 2016). In another study that examined resting-state EEG before and after medication treatment, lower pre-treatment activity in the oral precentral gyrus and the medial frontal gyrus in the beta band was associated with greater treatment response (Fontenelle et al., 2006).

#### 1.2.3. Positron emission tomography

Positron emission tomography (PET) can provide quantitative biological information in the body. Depending on the contrast agent used, PET can explore the distribution and expression of different substances in the human body. TSPO is responsible for the translocation of cholesterol from the outer mitochondrial membrane to the inner mitochondrial membrane, thus limiting the rate of neurosteroid biosynthesis, and the increase of TSPO levels after brain injury mainly occurs in the injured primary or secondary regions that express activated glial cells. Therefore, in OCD, researchers often use radioligands based on TSPO to study the synthesis of neurosteroids (Zhang et al., 2021).

### 1.3. The development trends of artificial intelligence research in obsessive-compulsive disorder

**Figure 1:**
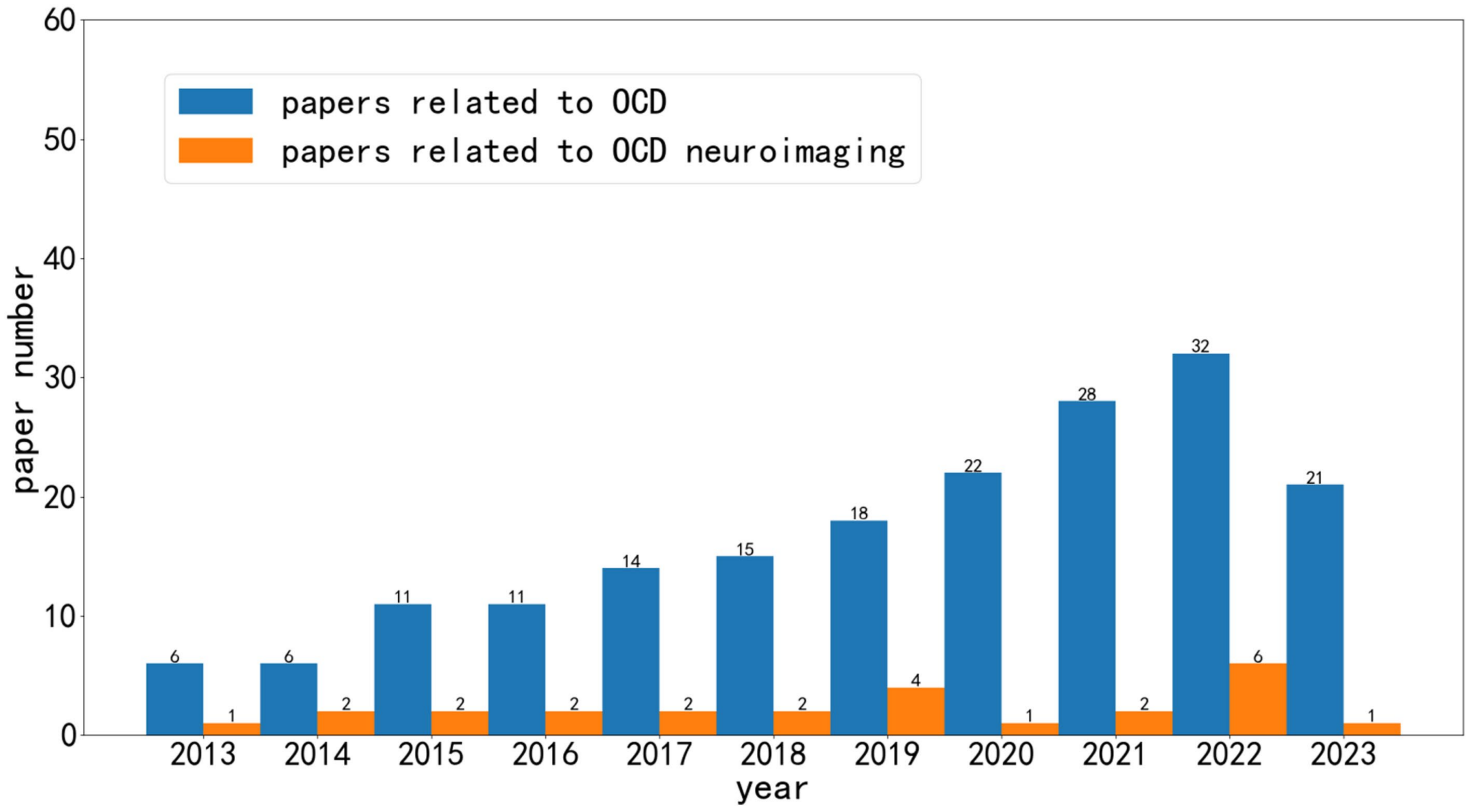
The literature on artificial intelligence for OCD from 2013 to 2022.

As shown in Figure 1, over the past decade, research related to artificial intelligence in the field of obsessive-compulsive disorder (OCD) has grown significantly. We can observe that AI research based on neuroimaging represents only a portion of this growth. A considerable number of articles are centered around scales related to OCD diagnosis, while some studies are based on social media and smart wearable devices.

### 1.4. Other reviews in this field

At present, a considerable number of researchers have noted the importance of neuroimaging and artificial intelligence in OCD, so there are some reviews to summarize the above research. A study published in 2022 summarized the progress of the Consortium of enhanced neuroimaging and genetics in OCD over the last 5 years through meta-analysis. However, the research based on the project mainly uses brain imaging to measure and statistically analyze various parts of the brain, and lacks large-scale artificial intelligence applications (Van den Heuvel et al., 2022). A 2022 study, which included 24 studies, looked at whether patients with neuropathy would benefit from cognitive therapy. In the study, however, obsessive-compulsive disorder accounted for only two (Vieira et al., 2022). And a 2023 review discussed machine learning in the core areas of mental illness based on brain imaging. However, the imaging methods included in this study only included MRI (Lv et al., 2023).

### 1.5. Paper search and reading

**Figure 2:**
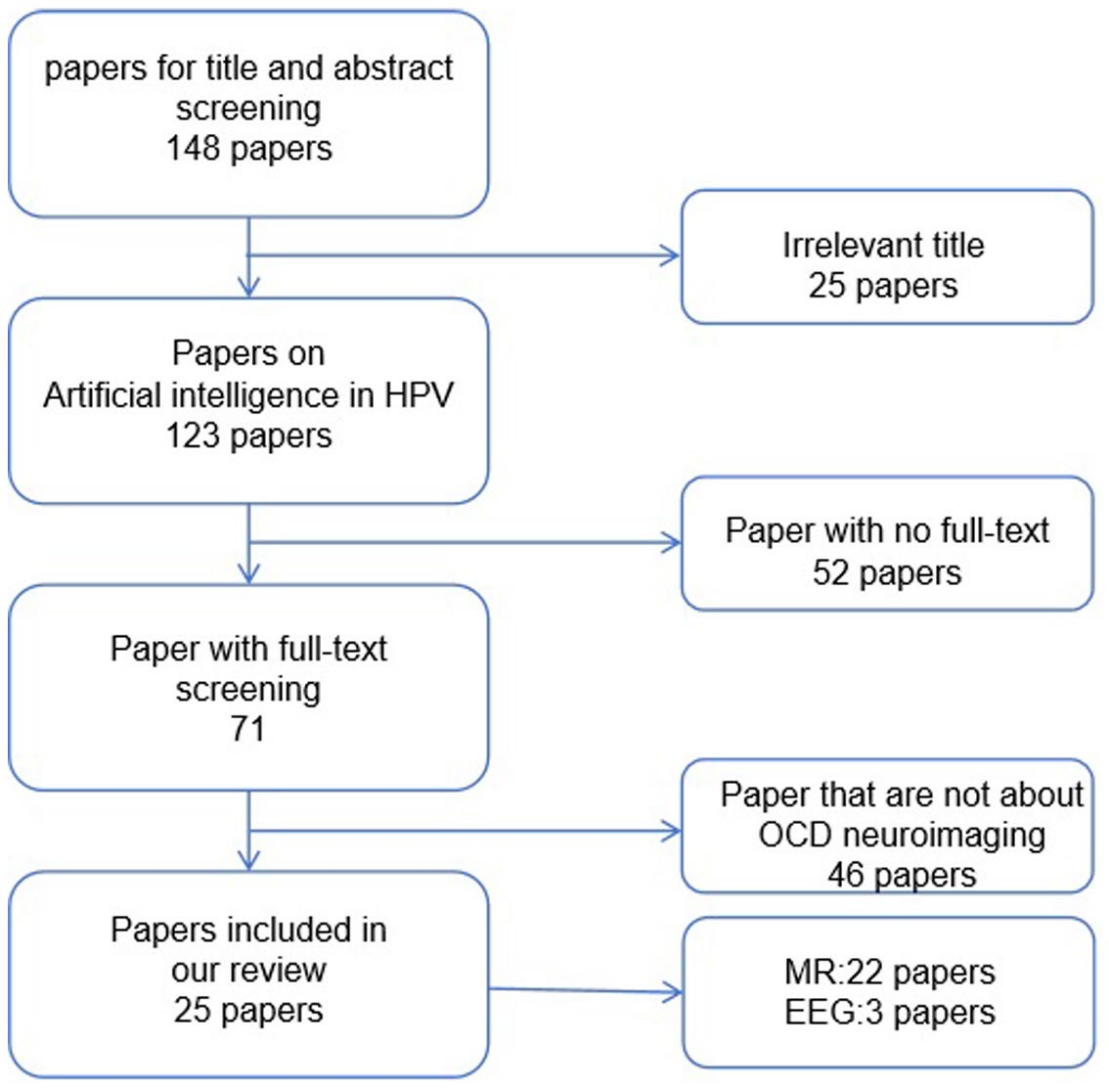
The selection process of papers in the review.

**Figure 3:**
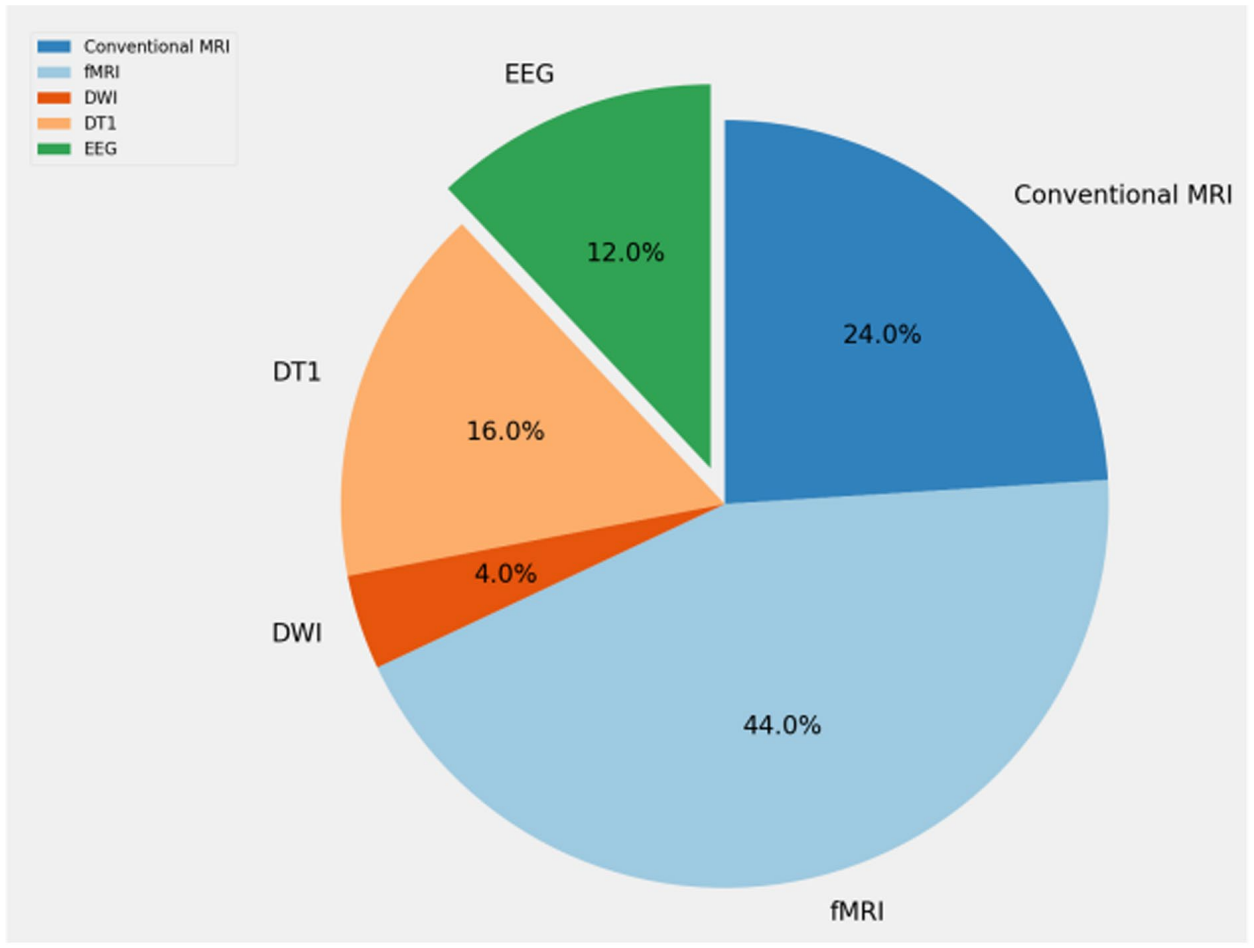
The percentage of each neuroimaging in this review.

As shown in Figure 2, we conducted a search using keywords such as “obsessive-compulsive disorder,” “artificial intelligence,” “machine learning,” and “deep learning” in PubMed. Considering the rapid developments in computer science, we limited our search to literature published within the last decade. We ended up with 148 articles. Subsequently, we began the screening process. Initially, we excluded articles that were not relevant to our research topic. Following that, we excluded articles related to other disorders that may share some symptoms with obsessive-compulsive disorder. Additionally, articles that did not utilize neuroimaging data for their research were excluded. Finally, we removed articles that presented highly redundant experimental methods. After these screenings, we included 25 studies related to the application of artificial intelligence in obsessive-compulsive disorder in this paper, as shown in Figure 3.

### 1.6. Article structure

The first section of the article primarily introduces the current state of diagnosis, treatment, and research related to obsessive-compulsive disorder (OCD) and artificial intelligence. The second section of the article focuses on the application of artificial intelligence in OCD based on various neuroimaging techniques. The third section of the article discusses the research potential of artificial intelligence based on other neuroimaging modalities in the field of OCD, as well as the application of artificial intelligence based on neuroimaging in other mental disorders.

## 2. Application of artificial intelligence in obsessive-compulsive disorder neuroimaging

### 2.1. MRI

**Figure 4:**
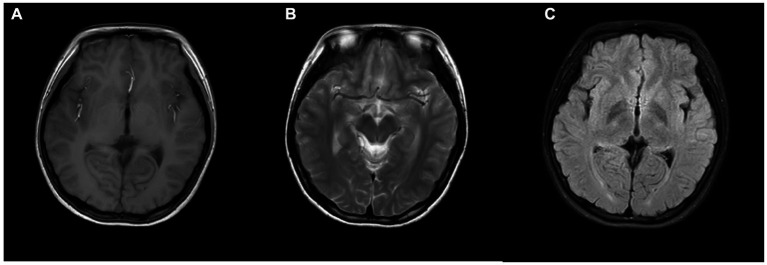
Picture of brain Conventional MRI, **(A)** T1, **(B)** T2, **(C)** FLAIR.

**Figure 5:**
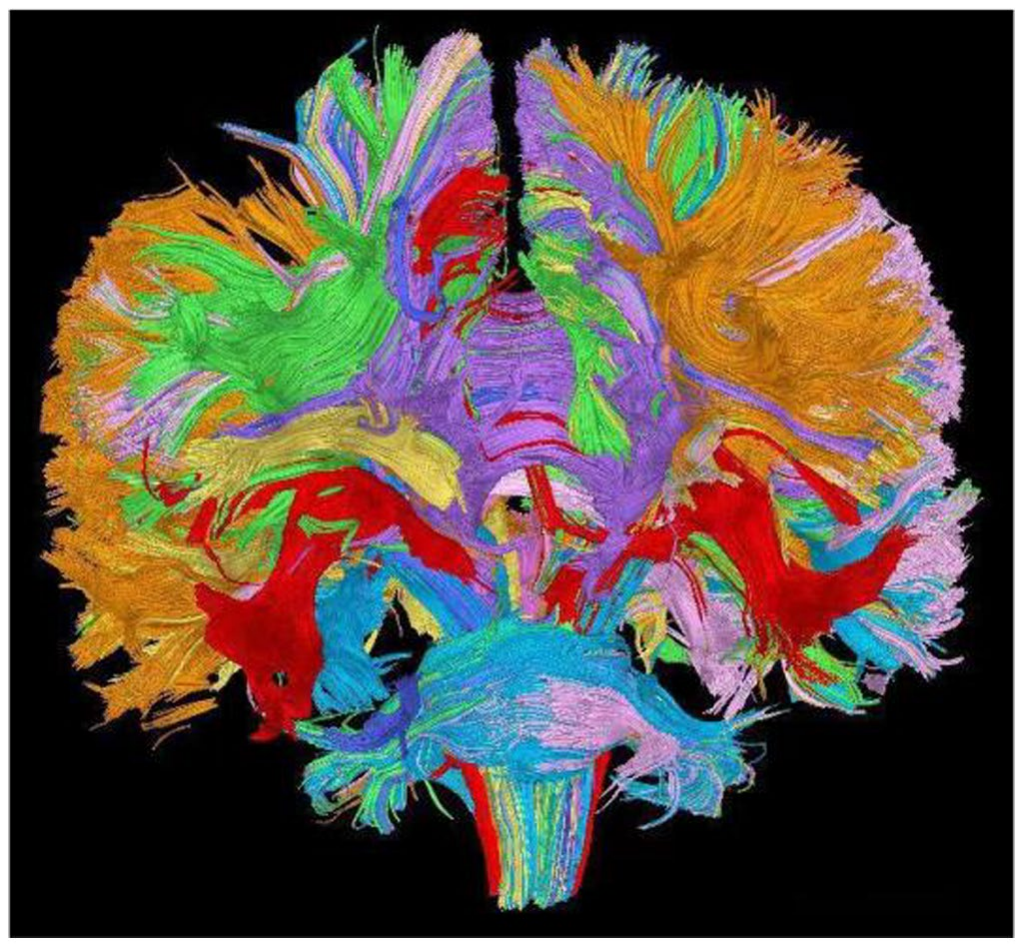
Picture of brain DTI.

**Figure 6:**
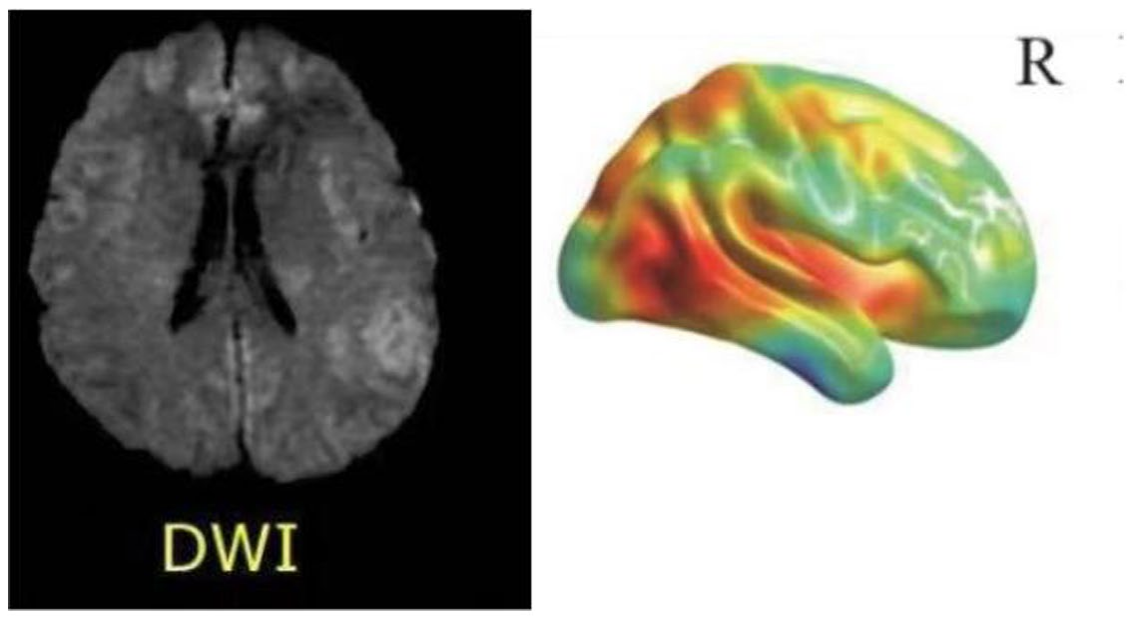
Picture of brain DWI.

MRI (Magnetic Resonance Imaging) stands as the most prevalent modality for cerebral examinations, offering high resolution to depict structural alterations within the cranium. As shown in Figure 4, a standard MRI examination should encompass T1, T2, and FLAIR sequences. Furthermore, MRI imaging possesses the versatility to create distinct imaging sequences by adjusting imaging protocol parameters, enabling a comprehensive multi-dimensional depiction of the brain. In examinations related to psychiatric disorders, DTI (Diffusion Tensor Imaging) and fMRI (Functional Magnetic Resonance Imaging) are commonly employed sequences, as shown in Figure 5. As shown in Figure 6, Diffusion Tensor Imaging (DTI), an evolution and refinement of Diffusion Weighted Imaging (DWI), presently stands as the sole non-invasive examination method capable of effectively observing and tracking brain white matter fiber bundles Blood Oxygen Level Dependent Functional Magnetic Resonance Imaging (bold-fMRI), an imaging technique developed since the 1990s, aims to explore brain function by detecting localized alterations in magnetic field properties arising from the discord between local increases in cerebral blood flow and oxygen consumption during neuronal electrical activity generation (Uğurbil, 2012). Therefore, in neurological examinations, MRI demonstrates efficacy in both organic and non-organic psychiatric disorders.

#### 2.1.1. Conventional MRI

In a 2013 study, researchers from the Psychiatry Institute of the University of São Paulo School of Medicine included 37 patients. They used MRI to mark regions of interest in both hemispheres, including the lateral and medial Orbitofrontal Cortex (OFC), Anterior Cingulate Cortex (ACC), Caudate Nucleus, Putamen, Pallidum, Thalamus, and Thalamus. These regions were designated as predictor variables. The severity of obsessive-compulsive disorder (OCD) symptoms in the patients was assessed using the Y-BOCS (Yale-Brown Obsessive-Compulsive Scale). Researchers utilized support vector regression (SVR) to construct a predictive model for OCD symptom severity. The results indicated a Pearson correlation coefficient of 0.49 (*p* = 0.002) between the Yale-Brown Obsessive-Compulsive Scale for Dimensional Assessment (DY-BOCS) total scores and the predicted symptom severity. For the total scores of the traditional Yale-Brown Obsessive-Compulsive Scale (Y-BOCS), the correlation coefficient was 0.44 (*p* = 0.006). The regions most informative for discrimination were the left medial orbitofrontal cortex and the left shell of the caudate nucleus. These findings suggest that machine learning methods such as SVR analysis can identify neurobiological markers from individual structural MRI datasets to predict the severity of OCD symptoms (Hoexter et al., 2013).

In a 2014 study, researchers recruited 86 people with OCD and 86 healthy people. In this study, all subjects underwent structural magnetic resonance imaging. They develop a new method of multivariable feature selection based on support vector machine, and compare it with mass univariate T-test selection and recursive feature elimination. The researchers believe that their method includes more brain regions, and each region contains more voxels, so it is a more comprehensive way to characterize OCD based on structural magnetic resonance of the head (Parrado-Hernández et al., 2014).

In a 2016 study, researchers recruited 33 people with OCD and 33 healthy people who served as a control group. The age, sex, and education levels of the patients and healthy people were basically the same. All participants underwent a 3-T structural magnetic resonance examination of the head. The researchers segmented the gray and white matter on the MRI images and measured the volume of the gray and white matter parts, respectively. The accuracy of SVM (75.76%) was lower than that of Gaussian regression (77.27%) on gray matter images, while the accuracy of SVM (81.82%) was lower than that of Gaussian regression (80.30%) on white matter images. This study suggests that the anatomical features of GM and WM may help distinguish patients with OCD from those with HCS. The SVM-based approach using WM volume showed the highest accuracy in revealing inter-group differences in the population, indicating its diagnostic potential in detecting highly enriched OCD patients at the individual level (Hu et al., 2016).

In a 2017 study, researchers recruited 74 people from the primary psychiatric services and bulletin of the Association of Tourette Syndrome and Obsessive-Compulsive Disorder (ASTOC) in Brazil, 38 of whom had OCD and 36 of whom were healthy. All subjects included in this study underwent a 1.5-T head magnetic examination. The researchers segmented the brain structure, labeling a total of 117 brain regions as features. A total of seven methods were used to screen features, and support vector machines were used for modeling. In distinguishing between healthy people and OCD, the best accuracy rate of the model is 71.64%; in distinguishing between healthy people and low level OCD, the best accuracy rate of the model is 77.12%; in distinguishing between healthy people and high level OCD, the best accuracy rate of the model is 71.86%; in distinguishing low level OCD and high level OCD, the best accuracy rate of the model is 71.86%. The optimal accuracy of the model is 73.68%. Although the database size is small, Chi Squared, Gain Ratio and Symmetrical Uncertainty are good feature selection methods (Trambaiolli et al., 2017).

In a 2021 study, 57 patients from Amsterdam UMC were enrolled to predict the clinical outcomes of patients undergoing deep brain stimulation. The researchers used Y-BOCS to assess the patients’ OCD levels. Due to the long time span of the patients included in this study, the imaging parameters of the imaging tests received by the patients were inconsistent. The researchers measured both gray and white matter in the subjects’ brains. The researchers modeled the model using 50-fold cross-validation and evaluated the model using AUC, accuracy, sensitivity, and specificity. The researchers found a significant association between Y-BOCS scores at 12-month follow-up and the volume of gray matter in the left nucleus, while none of the machine learning models built by support vector machines showed acceptable predictive power. Among them, the best model was established by the gray matter volume of the nucleus, with an accuracy of 57.30% (Liebrand et al., 2021).

In a 2022 study, researchers proposed a new anatomical subtype of OCD associated with gray matter. The researchers recruited 100 people with obsessive-compulsive disorder and 100 healthy controls. The obsessive-compulsive disorder patients were identified by two psychiatrists according to the fifth edition of the Diagnostic and Statistical Manual of Mental Disorders. All patients with OCD included no other psychiatric disorders. The included control patients not only had no personal history of mental illness, but also had no first-degree relatives with mental illness. In this study, all included subjects underwent magnetic resonance examination. The researchers measured brain regions based on CAT 12. The researchers used maps of 268 brain regions to characterise them and used a semi-supervised method called HYDRA to categorize people with OCD into subtypes. The researchers found that structural heterogeneity was higher in patients with OCD than in healthy patients. In OCD, researchers believe there are two distinct subtypes (Han et al., 2022).

#### 2.1.2. Functional Magnetic Resonance Imaging

In a 2015 study, researchers recruited 56 patients from Seoul National University Hospital. The individual structural covariance based on cortical surface and thickness was characterized and modeled using support vector machines (SVM) to explore its use as a biomarker to predict the therapeutic effect of serotonin reuptake inhibitors. In this study, all subjects underwent MRI, followed by SRI therapy for 4 months, and underwent clinical reassessment of Y-BOCS, HAM-D, and HAM-A compositions. Based on the percentage change in Y-BOCS score after 4 months, subjects with an increase of ≥35% in Y-BOCS score were considered to have responded to treatment, and all other subjects were considered to have not responded to treatment. The researchers modeled the cortex using support vector machines after extracting features from 148 regions. The accuracy of distinguishing between the therapeutic and non-therapeutic models was 89.0%, the accuracy of distinguishing between the therapeutic and healthy models was 95.6%, and the accuracy of distinguishing between the therapeutic and healthy models was 90.7% (Yun et al., 2015).

In a 2017 study, researchers collected 108 enrollees from Kyoto Prefectural University of Medicine, of whom 56 had OCD and 52 were healthy. Among these subjects, Y-BOCS was used to evaluate whether they had obsessive-compulsive disorder. After standard preconditioning of 140 regions of interest (ROI) covering the entire brain, the researchers evaluated the interregional FC for each participant and then obtained the FC matrix. The researchers built the classifier by combining two machine learning algorithms: SLR and L1-SCCA. The model created by the researchers could classify people with OCD and healthy people with 73 percent accuracy and an AUC of 0.81. The researchers also tested the model using an external test set, with an AUC of 70.1%. The researchers’ model was better at classifying patients who were taking medication than those who were not (Takagi et al., 2017).

In a 2018 study, researchers recruited a total of 42 people with OCD from UCLA, clinics, and other sources. All patients were diagnosed by a psychiatrist through YBOCS. All enrolled patients underwent 3T FMRI. The researchers extracted features from a total of 196 spherical ROIs and established a functional matrix. Finally, the researchers used support vector machine to establish a machine learning network based on default mode network and visual network, respectively, to predict the treatment effect. Among them, the accuracy of the model based on default mode network is 67.9%, and the accuracy of the model based on visual network is 70.0% (Reggente et al., 2018).

A study from 2019 involved the inclusion of 40 patients with Obsessive-Compulsive Disorder (OCD) and 38 gender, age, and education level-matched Healthy Controls (HCs), who underwent resting-state functional magnetic resonance imaging (fMRI) scans. The patients were recruited from the Fourth Affiliated Hospital of Qiqihar Medical University and the Qiqihar Mental Health Center in China. The researchers conducted an analysis on the imaging data using seed-based functional connectivity (FC) and Support Vector Machine (SVM). SVM classification analysis revealed that a combination of left Crus I to left superior medial prefrontal cortex (MPFC) connectivity and right Crus I to left central MPFC connectivity could be used to differentiate between OCD patients and controls. The sensitivity was 85.00%, specificity was 68.42%, and accuracy was 76.92%. This study underscores the contribution of cerebellum-default mode network (DMN) connectivity in the pathophysiology of OCD and provides new insights into OCD research (Lv et al., 2020).

In a 2019 study, researchers enrolled 54 people with OCD from Sichuan University’s West China Hospital and matched 54 healthy people. All participants included in the study were right-handed and native Chinese speakers. Y-BOCS was used to assess the degree of obsessive-compulsive disorder, HAMA was used to assess anxiety, and HAMD was used to assess depression. All subjects underwent 3T magnetic resonance imaging. Brain images obtained by the team were calculated for ALFF, fALFF, ReHo and FCS, respectively. Based on the above four calculations, the researchers used support vector machine modeling to classify patients and healthy people. The accuracy rate of the model based on ALFF was 95.37%, the accuracy rate of the model based on ReHo was 86.11%, the accuracy rate of the model based on fALFF was 82.41%, and the accuracy rate of the model based on FCS was 74.07%. However, the four models have different concerns. The main brain regions used for ALFF classification include left vmPFC, right dorsal lateral prefrontal cortex and bilateral insula; The main brain regions used by ReHo for classification include bilateral orbitofrontal cortex (OFC), left ACC, left putamen, and left precentral gyrus; The main brain regions used for fALFF classification include right superior frontal lobe, bilateral precentral gyrus, right superior temporal gyrus, anterior cingulate cortex (ACC), and left cuneus and left lingual gyrus; The main brain regions used for FCS classification include bilateral superior frontal lobe, left vmPFC, left ACC, left superior parietal, bilateral lingual gyrus, and right putamen (Bu et al., 2019).

In a 2019 study, researchers retrospectively recruited patients who were admitted to West China Hospital of Sichuan University from 2012 to 2015. The researchers included 68 people diagnosed with OCD and healthy people of similar age, sex, and education. In this study, all subjects underwent a 3 T magnetic resonance examination. The power spectrum is obtained by transforming the time series into the frequency domain. The square root is calculated at each frequency of the power spectrum, and the root mean square in the low frequency range (0.01 ~ 0.08 hz) is obtained. This average is defined as ALFF. fALFF is calculated as the ratio of power in the low frequency range to power in the entire frequency range (0–0.25HZ). Finally, the spatial falff map was normalized, and each voxel was divided by the mean value of the whole brain falff to obtain the spatial map “mfALFF.” Based on mfALFF, the researchers used a support vector machine to build a model that classified OCD patients and healthy people. The final accuracy of the model was 72%, and the model focused on the left superior temporal gyrus, right middle temporal gyrus, left superior marginal gyrus and parietal lobule (Yang et al., 2019).

In a 2021 study, researchers enrolled a total of 44 people with OCD and 25 healthy controls, all recruited through clinics and communities in Los Angeles. In this study, all subjects underwent a 3 T magnetic resonance examination. The researchers performed deconvolution to extract HRF parameters from fMRI data. HRF is characterized by three parameters - response height (RH), time-to-peak (TTP), and time-to-peak (FWHM). The researchers tested two hypotheses: (1) HRF is sensitive to neuropathology: individuals with OCD will exhibit HRF aberrations compared to HC at baseline; and (2) HRF is sensitive to treatment and predicts treatment response. Pre-treatment HRF predicted treatment outcome (OCD symptom reduction) with 86.4% accuracy, using machine learning (Rangaprakash et al., 2021).

In a 2022 study, researchers enrolled 188 patients with OCD who visited the Clinic of the National Institute of Mental Health & Neuro Sciences and 200 healthy people of similar age and sex. Due to incomplete imaging data or clinical data, the investigators excluded 13 patients and 25 healthy individuals, so a total of 175 obsessive-compulsive disorder patients and 175 healthy individuals were included in the study. The researchers used EMPaSchiz as a neural network model. EMPaSchiz extracted six resting brain FMRI features, including three region-based features and three connection-based features. The model was 80.3% accuracy, 82.7% sensitive, 79.2% precise, and 77.8% specific. The researchers compared this network with a model built using other features, confirming that EMPaSchiz had a better effect. EMPaSchiz, which uses schizophrenia data for transfer learning, has also achieved good results (Kalmady et al., 2022).

In a 2022 study, researchers enrolled 54 patients from the outpatient department of Anhui Medical University. Patients who meet the DSM-V diagnostic criteria for OCD are jointly diagnosed by two clinicians. The severity of a patient’s symptoms is diagnosed by YBOCS. All enrolled patients underwent 3T magnetic resonance imaging. The researchers constructed a whole-brain connection matrix based on 268 subregions from brain images and used Connectome-based predictive modeling to model the matrix. First, we successfully predicted compulsion scores, but not obsession sores for a negative functional network and a positive structured network. We then found that the functional connectivity of triple networks (SN, DMN, and FPN), specifically SN-FPN, is primarily helpful in predicting individual forcing severity, with SN being a key predictor in structural prediction networks. These results suggest that structural SN and triple network jointly contribute to forcing (Zhu et al., 2022).

In a study conducted in 2022, the researchers recruited 128 participants diagnosed with Obsessive-Compulsive Disorder (OCD), comprising both adults (aged 24–45 years) and adolescents (aged 12–17 years), as well as an unaffected control group (*n* = 64). Neurocognitive assessments included tests for cognitive interference and error processing. Unsupervised machine learning was employed by the researchers to identify subgroups or clusters within the OCD patient cohort based on task-based functional magnetic resonance imaging assessments. Their primary focus was on the activation patterns within three large-scale brain networks associated with cognitive control and performance monitoring, which are relevant to OCD. The researchers identified three patient clusters, reflecting a “normative” cluster that shared brain activation patterns with the unaffected control group and two other clusters, namely an “interference-dominant” cluster and an “error-dominant” cluster. These clusters were then further examined for associations with demographic and clinical characteristics. Following correction for false discovery rates, it was observed that the interference-dominant cluster exhibited significantly longer reaction times compared to the other patient clusters, although no other covariate differences were detected between the clusters. These findings enhance the precision of patient characterization, redefining previous neurobehavioral studies of OCD, and providing a starting point for neuroimaging-guided treatment selection (De Nadai et al., 2023).

In a 2023 study, researchers used a total of 1,024 obsessive-compulsive disorder patients and 1,028 healthy people from the ENIGMA-OCD consortium. All enrolled patients underwent either 1.5T or 3T magnetic resonance imaging. All researchers analyzed differences in resting state functional connectivity between OCD patients and healthy controls (HCS). We assessed population differences in whole-brain functional connectivity at the regional and network levels and investigated whether functional connectivity could be used as a biomarker to identify patient status at the individual level using machine learning analysis. Extract a Time series from 434 regions of interest using a combination of functional and structural maps. The linear support vector machine (SVM) model implemented in scikit-learn (v1.0.2, Python v3.9.5) was used for multivariate classification. Performance was assessed using 20 repeated stratified quintuples cross validation (CV) and measured as the mean area under the receiver operating characteristic curve (AUC). Eventually, the researchers created multiple models that classified normal and OCD patients. The overall performance of the models is low, but the significance is significant, and the AUC of different models is between 0.567 and 0.673. In contrast, adult patients are more easily distinguished from strong normal people than children patients. The drug patients were more easily distinguishable from the normal people than the non-drug patients. Researchers believe that people with OCD have extensive FC aberrations, low overall connectivity, and few hyperconnections. Notably, most of the important low connections lie within the sensorimotor network (Bruin et al., 2023).

#### 2.1.3. Diffusion Weighted Imaging

In a 2020 study, researchers recruited 176 Children from the Hospital for Sick Children and Holland Bloorview Kids Rehabilitation Hospital, 56 of whom had ADHD, 81 named autism spectrum disorder and 39 named obsessive-compulsive disorder. All patients were examined on the same 3 T MRI machine. We used similarity network fusion to measure cortical thickness, subcortical volume, white matter fraction anisotropy (FA), and behavior in these patients. The researchers used random forest modeling to classify patients by disease type. The Mean specificity performance of the final model was >80%, and Mean sensitivity performance was >62–75%. In this model, we found that cortical thickness in areas important for social or language-related behavior (inferior frontal gyrus, insula, inferior parietal cortex, temporal cortex) and executive function (upper and middle frontal gyrus), as well as inattention scores, were the main categorical features (Jacobs et al., 2021).

#### 2.1.4. Diffusion Tensor Imaging

In a 2014 study, researchers enrolled 28 people with OCD and 28 healthy people from the Mental Health Center of Sichuan University’s West China Hospital. All subjects underwent 3 T magnetic resonance examination and obtained DTI images. The researchers built a classification model based on support vector machines to distinguish between OCD patients and non-OCD patients. The model was verified using the leave-one method. In the established model, the accuracy rate is 84%. The researchers looked at Y-BOCS scores in patients and the distance of patients from the hyperplane in the SVM, and found that individuals with higher Y-BOCS scores tended to be further from the hyperplane, while individuals with lower impairment levels tended to be closer to the hyperplane, so it can be inferred that the model classifies patients and non-patients based on OCD symptoms measured by Y-BOCS. In this model, the brain regions mainly based on the classification model were bilateral prefrontal and temporal white matter, subfronto-occipital fasciculus, parietal frontal fasciculus, splenic corpus callosum and left middle cingulate fasciculus (Li et al., 2014).

In a 2016 study, researchers enrolled 56 patients from the Department of child and adolescent Psychiatry and Psychology at the Clinical Hospital of Barcelona. All patients had complete neuroimaging (including structural MRI and DTI), All underwent neuropsychological assessments (Wechsler Intelligence Scale, Wechsler Memory Scale, Verbal Fluency Test, Trail Making Test, Rey Complex Figure Test, and the Stroop Test) and genetic data testing (including rationale of candidate genes selection, single nucleotide polymorphism [SNP] selection criteria, genotyping methodology, and quality control). Entropy-based information gain (IG) measurements were used for feature selection and two supervised machine learning methods (SVM and NB) were used for modeling to identify patients’ OCD severity. The Accuracy of SVM is 0.96 in the internal verification of leave-one method. Sensibility: 0.94; Specificity: 1.00; Precision: 0.95; AUC: 0.98. The Accuracy of SVM is 0.69 on the external verification set. Sensibility: 0.71; Specificity: 0.67; Precision: 0.63; AUC: 0.75. The Accuracy obtained by NB in the internal verification of the remaining one method is 0.94; Sensibility: 0.87; Specificity: 0.89; Precision: 0.87; AUC: 0.88. The Accuracy obtained by NB on the external verification set is 0.65; Sensibility: 0.81; Specificity: 0.50; Precision: 0.75; AUC: 0.77. Therefore, the researchers suggest that SVM and NB may be useful in finding predictors of diagnosis in patients with early-onset OCD (Mas et al., 2016).

In a 2018 study, researchers enrolled a total of 48 OCD patients from the Department of Psychiatry at the First Affiliated Hospital of Kunming Medical University and 45 healthy people recruited through other channels. In this study, all subjects were right-handed Han Chinese aged 18 to 55 years. All obsessive-compulsive patients were assessed by Y-BOCS, and those with prominent symptoms of depression and anxiety were excluded. All subjects underwent a 3 T magnetic resonance examination and obtained DTI images. Grey matter, white matter and cerebrospinal fluid were segmented and smoothed. The model is established by support vector machine and verified by leave-one method. Four models were developed, with an accuracy of 72.04%, a Sensitivity of 70.83%, a Specificity of 73.33, and an AUC of 0.71. The accuracy of Specificity based on WMV was 61.29%, Sensitivity 64.58%, Specificity 57.78, AUC 0.61. The accuracy of FA-based Specificity was 80.65%, Sensitivity 81.25%, Specificity 80.00, AUC 80.00; The accuracy, Sensitivity, Specificity and AUC of MD were 77.42, 75.00%, 80.00, and 0.84 (Zhou et al., 2018).

In a 2019 study, researchers enrolled a total of eight people with obsessive-compulsive disorder. All patients underwent 3 T magnetic resonance imaging and all had DTI images. The researchers introduced Hamlets, a layered harmonic filter for learning tracks from diffusion MRI, into the DTI analysis. HAMLET is an algorithm that is able to map raw diffusion MRI data directly onto a directional graph of trace presence and direction. This method can automatically display the anatomical structure of slMFB (superolateral branch of the Medial Forebrain Bundle) after MRI is obtained (Coenen et al., 2019).

#### 2.1.5. Electroencephalogram

**Figure 7:**
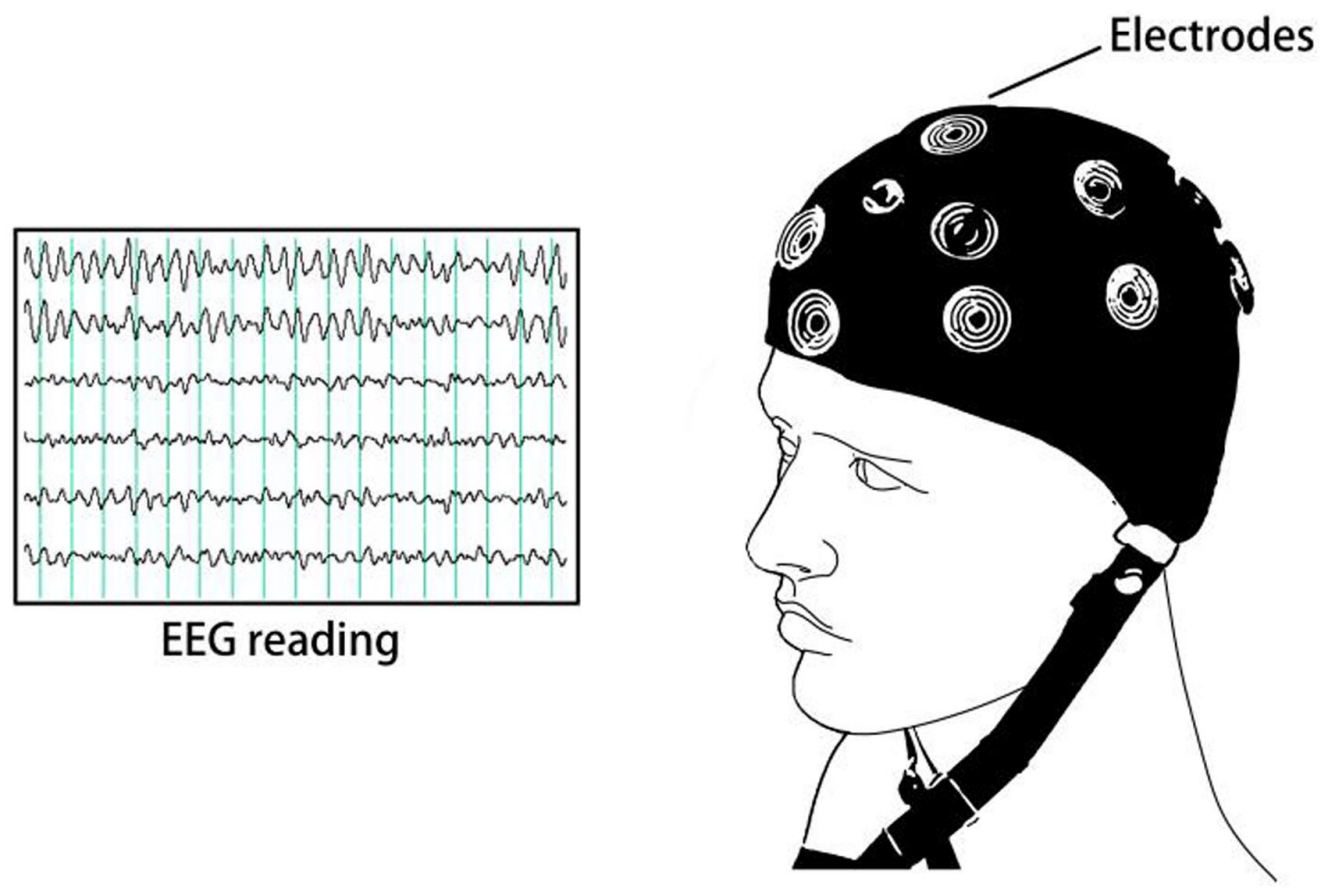
EEG schematic diagram.

Electroencephalogram (EEG) sensor measurements represent a temporal process that quantifies the sum of electrical activity occurring at a cortical location in terms of amplitude, as shown in Figure 7. This signal is rich in information that can be extracted using various techniques. Apart from being cost-effective and easy to implement, resting-state EEG offers the advantage of capturing dynamic changes in neuronal networks with high temporal resolution. Due to these advantages, EEG can be utilized for early diagnosis of psychiatric disorders and serve as a reliable biomarker. In high-risk individuals, EEG has been shown to exhibit high sensitivity (de Bock et al., 2020). Patients in a psychiatric high-risk state exhibit a higher prevalence of pathological abnormalities in their electroencephalograms (EEG) (Gschwandtner et al., 2009). In chronic mental illnesses, electroencephalograms (EEG) also hold significant clinical utility. Numerous studies have demonstrated the value of EEG in patients with schizophrenia, reaffirming its clinical relevance (Barros et al., 2021). In Alzheimer’s disease, electroencephalography (EEG) is also considered a potential tool for predicting cognitive decline in patients (Lopez et al., 1997).

In a study conducted in 2015, a classification of individuals with obsessive-compulsive disorder (OCD) was performed using single-channel complexity features (19 features) and inter-hemispheric dependency features (8 features) extracted from the data. This classification was carried out using a support vector machine. In this study, the model built using the prefrontal region of the brain had the highest accuracy of 85 ± 5.2% (Aydin et al., 2015).

In a 2021 study, the researchers enrolled patients with nine diagnoses of six diseases from the Seoul Metropolitan Government-Seoul National University (SMG-SNU) Boramae Medical Center. The study included a total of 945 patients, including 95 healthy people who served as controls. All subjects underwent EEG examination. These patients were classified using three machine learning algorithms and verified using 10x cross-validation. To select the model, the researchers compared the performance of SVM, RF, and EN in terms of AUC. EN showed the highest accuracy, with a mean AUC of 87.59 ± 7.92% across all diseases (SVM = 86.02 ± 8.89%, RF = 87.18 ± 8.08%) (Park et al., 2021).

In a 2023 study, researchers recruited 550 EEGs stored from the Uskudar University Cognitive Neuroscience Laboratory, 81 for bipolar disorder, 95 for attention deficit and hyperactivity disorder, 67 for depression, there were 34 obsessive-compulsive disorder, 75 opioid addiction, 146 post-traumatic stress disorder, 52 schizophrenia and 84 healthy individuals. The researchers used 80% of the data as the training set and 20% as the training set. The classification model was established using C5.0, random forest (RF), support vector machine (SVM) and artificial neural networks (ANN). In the established model, the highest accurate value is 0.841, which is obtained from the C5.0 model and the SVM model (Emre et al., 2023).

### 2.2. Summary

Table 1 shows the application of artificial intelligence in neuroimaging of obsessive-compulsive disorder included in this article. We show the year of publication, main researchers, data types used, and main methods used. Researchers tend to use MRI for research. fMRI is the data type most commonly used by researchers. EEG, which has less research, possibly due to the difficulty in processing EEG data, there are few sightings of effective information, and no studies have used PET/CT as experimental data, mainly due to the difficulty of data acquisition.

**Table 1:** Summary of papers on machine learning on OCD neuroimaging.

year	Research team	Data	Method	Algorithm	Result
2013	Marcelo Q Hoexter et al.	Conventional MRI	Machine learning	SVR	–
2014	Emilio Parrado-Hernández et al.	Conventional MRI	Machine learning	SVM	–
2014	Fei Li et al.	DTI	Machine learning	SVM	Accuracy: 84%
2015	Je-Yeon Yun et al.	fMRI	Machine learning	SVM	OCD-R vs. OCD-NR(accuracy): 89.0%/OCD-R vs. HC(accuracy): 95.6%/HC vs. OCD-NR(accuracy): 90.7%
2015	Serap Aydin et al.	EEG	Machine learning	SVM	Accuracy: 85 ± 5.2%
2016	Xinyu Hu et al.	Conventional MRI	Machine learning	SVM/GP	Gray matter(accuracy): 75.76% /77.27%; white matter(accuracy): 81.82%/80.30%
2016	Sergi Mas et al.	DTI	Machine learning	SVN/NB	SVM(accuracy): 96.00%/NB(accuracy): 94.00%
2017	Lucas R. Trambaiolli et al.	Conventional MRI	Machine learning	FS	Controls vs. All OCD(accuracy): 71.64%/Controls vs. Low OCD(accuracy): 77.12%/Controls vs. High OCD(accuracy): 71.86%/Low OCD vs. High OCD(accuracy): 73.68%
2017	Yu Takagi et al.	fMRI	Machine learning	SLR + L1-SCCA	Accuracy: 73.00%
2018	Nicco Reggente et al.	fMRI	Machine learning	SVM	Fault mode network(accuracy): 0.67.9%/visual network(accuracy): 70.00%
2018	Cong Zhou et al.	DTI	Machine learning	SVM	Accuracy: 80.65%
2019	Dan Lv et al.	fMRI	Machine learning	SVM	Accuracy: 76.92%
2019	Xuan Bu et al.	fMRI	Machine learning	SVM	Accuracy: 95.37%
2019	Xi Yang et al.	fMRI	Machine learning	SVM	Accuracy: 72.00%
2019	Volker A. Coenen et al.	DTI	Machine learning	HAMLET	–
2020	Grace R. Jacobs et al.	DWI	Machine learning	RF	Sensitivity>62–75%/specificity>80%
2021	Luka C. Liebrand et al.	Conventional MRI	Machine learning	SVR	Accuracy: 57.30%
2021	Su Mi Park et al.	EEG	Machine learning	EN/SVM/RF	EN(AUC): 87.59 ± 7.92%/SVM(AUC): 86.02 ± 8.89%/RF(AUC): 87.18 ± 8.08%
2022	Shaoqiang Han et al.	Conventional MRI	Machine learning	HYDRA	–
2022	Sunil Vasu Kalmady et al.	fMRI	Deep learning	EMPaSchiz	Accuracy: 80.30%
2022	Chunyan Zhu et al.	fMRI	Machine learning	CPM	–
2022	Alessandro S. De Nadai et al.	fMRI	Machine learning	–	–
2022	D Rangaprakash et al.	fMRI	Machine learning	-	Accuracy: 86.40%
2022	İlkim Ecem Emre	EEG	Machine learning	SVM	Accuracy: 84.10%
2023	Willem B. Bruin et al.	fMRI	Machine learning	SVM	AUC: 0.673

## 3. Discussion

### 3.1. Existing methods

As artificial intelligence continues to advance in the field of medicine, machine learning is playing an increasingly important role in the risk prediction, diagnosis, and treatment of Obsessive-Compulsive Disorder (OCD). Currently, in AI-related research, there are two main types of neuroimaging examinations for OCD: MR (Magnetic Resonance) and EEG (Electroencephalogram). Undoubtedly, MR is the most widely utilized neuroimaging modality. This is primarily due to MR’s non-invasive ability to capture the pathological and physiological abnormalities in the core brain, enabling the application of tailored early interventions based on pathology. Current research indicates that in the early stages of psychiatric disorders, changes in brain structure such as global or gray matter reduction can be identified from patients’ head scans. Meanwhile, MR’s various imaging modalities provide researchers with more comprehensive medical information, allowing the construction of more robust models. Techniques like DTI (Diffusion Tensor Imaging) can be used to examine white matter microstructure, thus quantifying the directionality and coherence of water diffusion. Resting-state functional MRI (rfMRI) studies have identified abnormal functional connectivity (FC) in OCD. Another study based on DWI (Diffusion Weighted Imaging) has suggested that the pathophysiology of OCD may involve not only abnormalities in the myelination status of the cortico-striato-thalamo-cortical circuit but also in the myelination status of posterior brain regions and temporal areas (Watanabe et al., 2018). Another significant examination modality is EEG (Electroencephalogram). Resting-state EEG is a simple, cost-effective examination method, thus it is widely favored for the assessment of various neurological conditions. Resting-state EEG can capture rapid dynamic changes in neural network activity with high temporal resolution, and as such, is considered capable of identifying biomarkers for numerous psychiatric disorders (Mikanmaa et al., 2019).

### 3.2. Potential methods in obsessive-compulsive disorder neuroimaging

As mentioned earlier, artificial intelligence in neuroimaging has made significant strides in the context of Obsessive-Compulsive Disorder (OCD). However, in the realm of medical imaging, the application of artificial intelligence remains largely limited to MR (Magnetic Resonance) and EEG (Electroencephalogram) due to their widespread availability. Additionally, some clinical practitioners have turned to PET (Positron Emission Tomography) for diagnosis and treatment in neurological disorders. PET’s advantage lies in its ability to assess neurotransmitters at the molecular level. Radiopharmaceuticals can bind to specific targets, reflecting the patient’s brain metabolism and elucidating the mechanisms underlying disease onset and progression. A study has indicated that in OCD, certain metabolism-related biomarkers such as N-acetylaspartate (NAA) and choline (Cho) may exhibit abnormal concentrations. The 18 kDa translocator protein (TSPO), formerly known as the peripheral benzodiazepine receptor, is primarily located in the outer mitochondrial membrane of steroid-producing cells. Brain TSPO expression is relatively low under physiological conditions but is upregulated in response to neuroglial cell activation. TSPO can be used to detect various neuroinflammatory conditions, and as such, TSPO is associated with the pathogenesis and progression of numerous neuropsychiatric and neurodegenerative diseases. Single Photon Emission Computed Tomography (SPECT) also holds a significant position in neuroimaging examinations for OCD patients. A SPECT-based study reported reduced availability of dopamine transporter protein in the striatum and decreased availability of serotonin transporter protein in the thalamus and midbrain, achieved through the use of SPECT scans and [123I]β-CIT, which labels dopamine transporter (DAT) and serotonin transporter (SERT) (Hesse et al., 2005). Furthermore, certain organic changes that lead to psychiatric disorders can be examined using CT (Computed Tomography). In fact, in neuroimaging research, progress has been made in all imaging modalities that can provide insights into a patient’s brain condition. These OCD-related biomarkers are identifiable through artificial means. Therefore, the primary limitation hindering researchers from building models based on other imaging methods is the lower prevalence of these alternative imaging techniques in OCD and the difficulty in gathering a sufficient number of patients.

### 3.3. Application of artificial intelligence in neuroimaging of other mental disorders

#### 3.3.1. Schizophrenia

Schizophrenia is a chronic and complex neurological disorder that affects both social and cognitive functioning. It is characterized by the presence of positive symptoms (e.g., hallucinations and delusions), negative symptoms (e.g., blunted affect), and cognitive deficits. Approximately 75% of patients experience auditory hallucinations, with auditory hallucinations being the most common (Jauhar et al., 2022). For the same reasons as in obsessive-compulsive disorder, EEG and MRI remain the predominant neuroimaging modalities in the context of artificial intelligence research related to schizophrenia. In a study conducted in 2022, researchers utilized an openly accessible Kaggle dataset to investigate alterations in electroencephalographic patterns during auditory processing and their potential in discriminating between individuals with schizophrenia and healthy controls (Roach, 2021); (Barros et al., 2022). In a study conducted in 2020, head magnetic resonance (MR) images of individuals diagnosed with schizophrenia were acquired from multiple centers. A three-dimensional convolutional neural network was established to classify individuals into groups of those with schizophrenia and healthy controls (Oh et al., 2020). In a study conducted in 2020, researchers proposed an innovative multi-level convolutional neural network that effectively amalgamated diverse neuroimaging-derived features. This network demonstrated effectiveness in discriminating between individuals with schizophrenia and those with autism spectrum disorders (Du et al., 2020).

#### 3.3.2. Depression

Depression is a highly prevalent mental disorder and is gradually becoming one of the leading causes of global disease burden. Given its rapidly increasing prevalence and significant impact on individuals’ personal lives and interpersonal relationships, depression can lead to social withdrawal and impose a substantial burden on public health. The COVID-19 pandemic has further exacerbated the mental health challenges faced by the public. Magnetic resonance imaging (MRI) remains one of the primary imaging modalities for depression. In a study conducted in 2023, structural MRI images were utilized to predict aspects of late-life depression, including anhedonia, suicidal tendencies, appetite, sleep disturbances, and anxiety (Cao et al., 2023). An EEG-based automated diagnostic system for depression has been suggested for early and accurate detection of mood disorders (Uyulan et al., 2022). In addition to artificial intelligence research in the field of neuroimaging mentioned above, studies analyzing patients’ voices have also become a focal point in the diagnosis of depression due to the often pronounced changes in energy, communication abilities, and emotions seen in individuals with this condition. In a study conducted in 2023, researchers employed pre-trained large-scale language models and parameter-efficient fine-tuning techniques to establish a neural network model capable of assessing the severity of depression in patients based on their spoken text (Lau et al., 2023). In another study conducted in 2022, researchers collected demographic information and acoustic data from 56 Mandarin-speaking elderly individuals with Major Depressive Disorder (MDD). They developed a deep learning model to diagnose early-onset late-life depression using raw voice signals recorded by patients on their smartphones (Lin et al., 2022).

#### 3.3.3. Alzheimer’s disease

**Figure 8:**
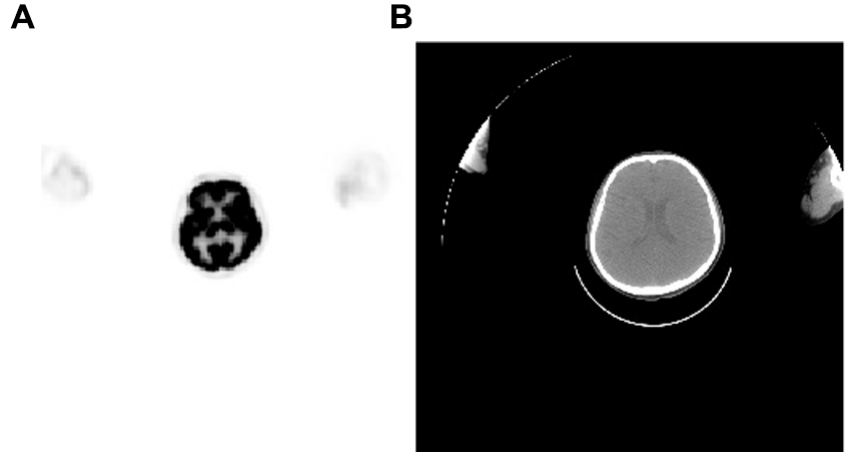
A sample of brain PET/CT, **(A)** PET, **(B)** CT.

Alzheimer’s disease (AD) is a progressive brain disorder and a critical global health, public health, and population health concern. Progressive memory loss and cognitive impairment are the primary features of Alzheimer’s disease. In fact, early diagnosis of Alzheimer’s disease heavily relies on neurocognitive tests, brain imaging, and cerebrospinal fluid analysis. Therefore, unlike other psychiatric disorders, access to brain imaging data for Alzheimer’s disease patients is more readily available. As of 2023, numerous studies have been conducted using MRI for the diagnosis of Alzheimer’s disease. In a study conducted in 2022, a novel model was developed, which utilized brain tissue segmentation from MRI data. This model employed a combination of Extreme Gradient Boosting (XGBoost) and Support Vector Machine (SVM) techniques to classify Alzheimer’s disease based on segmented brain tissues (Tuan et al., 2022). Simultaneously, there is a substantial body of research focused on diagnosing Alzheimer’s disease based on EEG (Electroencephalogram) data (Bi and Wang, 2019; Alessandrini et al., 2022). Furthermore, due to the accumulation of hyperphosphorylated and pathologically misfolded tau proteins being a primary and most common hallmark of Alzheimer’s disease (AD), there is also research exploring early detection of the prodromal stages of AD using tauPET, as shown in Figure 8 (Jo et al., 2020).

#### 3.3.4. Post-traumatic stress disorder

Trauma exposure can lead to a range of psychiatric disorders, including depression, anxiety disorders, bipolar mood disorders, personality disorders, psychosis, and trauma-related disorders, particularly post-traumatic stress disorder (PTSD). PTSD is a pathological diagnosis characterized by a history of trauma exposure and the presence of symptoms lasting for at least 1 month. These symptoms stem from at least one of four clusters: intrusion, avoidance, negative alterations in mood and cognition, and arousal and reactivity (Compean and Hamner, 2019). In a study conducted in 2023, an EEG-based approach was employed, integrating Convolutional Neural Networks (CNN) and Long Short-Term Memory (LSTM) networks in a multi-input CNN-LSTM architecture. This deep learning model demonstrated high accuracy in identifying fear emotions from physiological signals (Masuda and Yairi, 2023). In another study utilizing resting-state functional MRI (rs-fMRI), data from 91 individuals diagnosed with post-traumatic stress disorder and 126 trauma-exposed individuals without PTSD were included. This research employed a combination of deep learning and graph theory-based brain features to detect post-traumatic stress disorder at the individual level using rs-fMRI data (Zhu et al., 2021).

#### 3.3.5. Anxiety disorder

Anxiety disorders are a common group of mental health disorders that are often characterized by excessive arousal, excessive fear and worry that are disproportionate to actual threats or dangers, and significantly interfere with normal daily functioning (Olthuis et al., 2016). Anxiety disorders often combine with multiple mental disorders, such as mood disorders, substance abuse, or personality disorders (Andrews et al., 2001; Olatunji et al., 2007; Barrera and Norton, 2009). There are many types of anxiety disorders, including social anxiety disorder, panic attacks, generalized anxiety disorder, agoraphobia, dissociative anxiety disorder, and selective mutism. In a 2023 study, researchers used data from the IMAGEN database to incorporate patient MRI to assess anxiety in adolescents. Using three machine learning methods, logistic regression (LR), support vector machine (SVM), and Random forest (RF), the researchers built models to demonstrate the correlation between gray matter volume and anxiety (Chavanne et al., 2023). In a 2022 study, researchers used EEG data to classify people with a wide range of anxiety disorders and healthy people. The accuracy of the model was 97.83 ± 0.40%, sensitivity 97.55 ± 0.31%, specificity 97.78 ± 0.36% of specificity, and F1 97.95 ± 0.17% (Shen et al., 2022).

## 4. Conclusion

With the advancement of medicine, clinical practitioners have gained a deeper understanding of numerous psychiatric disorders. Mental illnesses, once stigmatized as “madness,” are gradually being accepted as manageable or treatable conditions. For healthcare professionals and patients alike, quantifying the diagnosis of mental disorders serves multiple purposes. On one hand, it aids in demystifying the enigma surrounding mental illness, thereby reducing potential discrimination. On the other hand, it facilitates more precise diagnoses and personalized treatment strategies. Although the application of artificial intelligence in neurological disorders is still in its infancy, some researchers are content with extracting interpretable biomarkers. Nevertheless, this field holds significant promise. AI based on neuroimaging possesses the capability to identify subtle alterations in mental illnesses by extracting complex and abstract features, surpassing diagnostic capabilities rooted solely in clinical experience. In this article, we discuss the application of artificial intelligence in neuroimaging of individuals with Obsessive-Compulsive Disorder (OCD). Most of the studies included in this article are based on brain atlases. We believe that extracting interpretable features based on brain atlases is an important research direction in the field of psychiatric neuroimaging. Therefore, we consider this to be highly beneficial for researchers in the field of mental disorders conducting quantitative studies on psychiatric illnesses. The application of artificial intelligence in OCD has two important implications. First of all, AI can directly build diagnosis and treatment prediction models for patients from images and medical record information, which not only reduces the workload of clinicians, but also provides important support for clinical work. Secondly, in the process of interpreting or feature screening the machine learning model established by neuroimaging, the occurrence and development principle of OCD is actually explored. Researchers can further deepen their understanding of OCD by identifying differences in neuroimage performance associated with OCD. Furthermore, the widespread adoption of neuroimaging examinations across various types of psychiatric disorders, coupled with comprehensive, systematic, and long-term follow-up data, may pave the way for the development of large-scale AI models in this field.

## Author contributions

XL: Project administration, Writing – original draft. QK: Investigation, Writing – original draft. HG: Visualization, Writing – original draft.
